# Editorial: Molecular mechanism of neuroimmune modulation and synaptic plasticity in acute and chronic pain, volume II

**DOI:** 10.3389/fnmol.2024.1484826

**Published:** 2024-09-12

**Authors:** Shunmei Lu, Yuxin Zhang, Zilong Wang, Linlin Zhang, Xin Zhang

**Affiliations:** ^1^Department of Anesthesiology, The Affiliated Wuxi People's Hospital of Nanjing Medical University, Wuxi People's Hospital, Wuxi Medical Center, Nanjing Medical University, Wuxi, Jiangsu, China; ^2^SUSTech Center for Pain Medicine, School of Medicine, Southern University of Science and Technology, Shenzhen, China; ^3^Department of Anesthesiology, Tianjin Medical University General Hospital, Tianjin, China; ^4^Tianjin Research Institute of Anesthesiology, Tianjin, China; ^5^Center for Translational Pain Medicine, Department of Anesthesiology, Duke University Medical Center, Durham, NC, United States

**Keywords:** pain, spinal GAT-1, TRPV1, ion channels, synaptic plasticity

Pain signals are conveyed to the central nervous system through specific receptors and nerve fibers, culminating in the perception of pain and influencing various biological systems. Recent research has increasingly concentrated on the molecular mechanisms and signaling pathways underlying both acute and chronic pain, thereby identifying novel potential therapeutic targets for future interventions. The growing interest among researchers in this field underscores its substantial potential to advance clinical pain management. This article provides a comprehensive overview of the latest developments in this area of study.

The GABAergic system is a vital component of neural circuits, serving as the primary inhibitory neurotransmitter and exhibiting widespread expression in spinal cord interneurons involved in sensory information processing. A reduction in inhibitory GABAergic neurotransmission can lead to disinhibition of excitatory transmission, resulting in heightened sensitivity to stimuli and the manifestation of spontaneous pain. Pradier et al. conducted a time-dependent study on GABA synthesis and uptake within a surgical incision pain model, aiming to elucidate the mechanisms and functional patterns of GABAergic transmission during the postoperative pain process. The researchers employed intrathecal administration of a specific antagonist (NO711) to inhibit spinal GAT-1, subsequently observing alterations in pain phenotypes and quantifying the expression of key proteins. Their findings indicate that GAT-1 plays a critical role in modulating spinal GABAergic signaling in the spinal dorsal horn shortly after incision, contributing to the evoked pain phenotype. Increased expression of GAT-1 leads to enhanced GABA uptake from the synaptic cleft, thereby reducing tonic GABAergic inhibition at the post-synapse. Inhibition of GAT-1 transiently restored this balance and alleviated the evoked pain phenotype. This study provides compelling evidence for the pivotal role of GABAergic signaling in modulating evoked pain responses and identifies GAT-1 as a promising target for perioperative pain management following incision injury.

TRP channels are critical molecular components involved in both acute inflammation and chronic pain conditions. Among them, the transient receptor potential vanilloid subtype 1 (TRPV1) channel is one of the most extensively studied mechanisms in peripheral neuropathic pain (NeuP) research, owing to its widespread expression in neuronal cells and its pivotal role in pain perception and modulation. In a comprehensive review by Gao et al., the authors provide a systematic overview of TRPV1, with particular emphasis on its role and recent research advancements in NeuP. The review meticulously examines the topic across six key areas: the structure and expression of the TRPV1 channel, mechanisms underlying NeuP, the functions of TRPV1 in pain regulation, the role of TRPV1 in NeuP mechanisms, the association between TRPA1 and TRPV1, and potential drug targets. The authors conclude that TRPV1 plays a dual role in peripheral NeuP, functioning as a “switch” for pain through its sensitization and desensitization processes. Their findings suggest that inhibiting TRPV1 channels can significantly reduce mechanical hypersensitivity and pain. Clinically, capsaicin, a TRPV1 agonist, alleviates pain by inducing receptor desensitization, while TRPV1 antagonists and siRNA targeting TRPV1 demonstrate promising results in preclinical studies.

Cancer-induced bone pain (CIBP), resulting from bone metastasis, is one of the most prevalent and challenging conditions to manage, with current treatments predominantly relying on opioids, which are associated with significant side effects. Ion channels on biological cell membranes are integral to various physiological processes, including pain signaling within the nervous system. In recent years, there has been growing interest in the role of ion channels in chronic pain, particularly within the context of CIBP. To enhance the understanding of potential therapeutic applications, Lu et al. explored the functional mechanisms of various ion channels, as well as the peripheral and central mechanisms involved in CIBP, alongside their clinical applications. The authors affirmed that ion channels play a critical role in CIBP, contributing to the transmission and modulation of pain signals, with their dysregulation potentially leading to the development of chronic pain. The study suggests that targeting multiple mechanisms involved in CIBP may be more effective than approaches focused on a single ion channel, offering new avenues for future therapeutic strategies in cancer pain management.

The review article by Li et al. focuses on the analgesic mechanisms of acupuncture for radicular pain, a condition with a complex pathophysiology that often leads to suboptimal clinical outcomes and significantly impacts patients' quality of life. While surgical treatment can provide rapid symptomatic relief, long-term outcomes do not significantly differ between surgical and conservative approaches, and pharmacologic treatments are often accompanied by side effects that hinder patient compliance. Acupuncture, an ancient Chinese therapy, is widely utilized for various types of pain, including lumbar and cervical radicular pain, as well as radicular pain associated with spinal stenosis. Despite its clinical efficacy, the precise mechanisms through which acupuncture alleviates radicular pain remain largely unclear. The review identifies four primary mechanisms of acupuncture's action: relieving mechanical compression of nerve roots, exerting anti-inflammatory effects, modulating spinal synaptic plasticity, and influencing brain regions involved in pain perception and processing. Although acupuncture demonstrates significant effects on radicular pain, current studies do not establish a direct causal relationship between acupuncture and pain relief. Methodological rigor is crucial in evaluating acupuncture's efficacy and elucidating its analgesic mechanisms. Present challenges include small sample sizes and a lack of standardized measures in existing studies. Consequently, advancing our understanding of acupuncture's analgesic mechanisms will require well-designed clinical trials with randomized controlled designs, larger sample sizes, and standardized outcome measures to mitigate potential confounders and provide robust evidence-based conclusions.

In conclusion, this Research Topic provides a comprehensive overview of the ongoing efforts to elucidate the mechanisms underlying post-surgical pain, peripheral NeuP, CIBP, and radicular pain. We hope that this Research Topic will be valuable to researchers seeking to deepen their understanding of the molecular mechanisms involved in neuroimmune modulation and synaptic plasticity in both acute and chronic pain, while also identifying potential therapeutic targets for future interventions.

